# Dr. Davis's Report of the Universal Dispensary

**Published:** 1817-10

**Authors:** J. B. Davis

**Affiliations:** 103, Great Surrey Street, Blackfriars


### Medico-Chirurgical

### JOURNAL AND REVIEW.

VOL. IV.] OCTOBER, 1817. [NO. 22.

### PART I.

### ORIGINAL COMMUNICATIONS.

——— quid utile ———

### To Dr. SHEARMAN.

DEAR SIR,

A Correct return of the diseases which have prevailed among children during the last year, taken from the cases concentrated at the Universal Dispensary for the Infant Poor, will, I doubt not, be deemed by the medical public a valuable record in the Annals of Medicine.

Under this impression, I have considered it a duty to draw up the following Report upon the cases which have come under my care, as the physician of that Institution: and should such an account, which has faithfully been prepared from authentic sources, appear to you a desirable acquisition, you will, I may venture to conclude, feel a pleasure in circulating the same, through the medium of your much admired and invaluable Journal.

I am,
Dear Sir,
Your's faithfully,
J. B. DAVIS, M. D.

103, Great Surrey Street, Blackfriars.
August 19, 1817.

*List of Cases received under the Care of the Physician at the Universal Dispensary for Children, St. Andrew's Hill, Doctors' Commons, London; from January* 30 *to July* 31, 1817.

| DISEASES. | Number of Cases received in each Month | | | | | | Total Number |
|---|---|---|---|---|---|---|---|
| | Feb. | Mar. | April. | May. | June. | July. | |
| Febris - - - - - - - - - - - | 2 | 4 | 4 | 2 | 1 | 12 | 25 |
| Febris catarrhalis epidem. | 14 | 4 | 7 | 1 | | | 26 |
| Febris post morbillos - - - | | | 3 | 5 | 6 | 7 | 21 |
| Febris remittens et hectica | 6 | 22 | 5 | 16 | 14 | | 63 |
| Febris periodica - - - - - | 1 | | | | | | 1 |
| Febris quotidiana - - - - | | 1 | 2 | | | | 3 |
| Phrenitis - - - - - - - - - | | | 4 | | 11 | | 15 |
| Cynanche tonsillaris - - - | 10 | 10 | | | 1 | | 21 |
| Cynanche parotidea - - - | | 2 | 11 | 12 | | 2 | 27 |
| Pneumonia - - - - - - - - | | 7 | 2 | 1 | 10 | 1 | 21 |
| Scarlatina - - - - - - - - - | | | | | 8 | 6 | 14 |
| Morbilli - - - - - - - - - - - | 13 | | 11 | 4 | 1 | 8 | 37 |
| Varicella - - - - - - - - - | 1 | 3 | | | 2 | | 6 |
| Urticaria - - - - - - - - - | 7 | | | 11 | | | 18 |
| Rheumatismus acutus - - | | 8 | | 10 | | | 18 |
| Epistaxis - - - - - - - - - | | | | | 1 | | 1 |
| Enteritis - - - - - - - - - - | | | 1 | | 7 | 10 | 18 |
| Dysenteria - - - - - - - - - | | | 1 | 1 | 2 | 1 | 5 |
| Diarrhœa - - - - - - - - - - | 7 | 15 | 10 | 3 | 2 | 5 | 42 |
| Cholera - - - - - - - - - - - | 2 | 1 | 7 | 1 | | 2 | 13 |
| Pertussis - - - - - - - - - - | 4 | 12 | 11 | 6 | 4 | 7 | 44 |
| Tussis - - - - - - - - - - - - | | 8 | 8 | 2 | 10 | 12 | 40 |
| Tussis cum dyspnœa - - - | | 1 | 1 | 1 | | | 3 |
| Dyspnœa - - - - - - - - - | 2 | 1 | | | | 2 | 5 |
| Phthisis - - - - - - - - - - | 1 | | 1 | | | | 2 |
| Hæmoptoe - - - - - - - - | 1 | 1 | | | | | 2 |
| Convulsiones - - - - - - - | 9 | 2 | 4 | 7 | 5 | 2 | 29 |
| Chorea - - - - - - - - - - - | 2 | 2 | 3 | | 1 | | 8 |
| Dolor capitis - - - - - - - | 3 | 4 | 2 | 5 | 3 | | 17 |
| Vomitiones - - - - - - - - | 2 | 1 | | 5 | 4 | 2 | 14 |
| Odontalgia - - - - - - - - | | 1 | 1 | | | | 2 |
| Otalgia - - - - - - - - - - - | | | | 1 | | | 1 |
| Dolor capitis periodicus - | | | | 3 | | | 3 |
| Surditas - - - - - - - - - - - | | | 1 | | | | 1 |
| Tormina - - - - - - - - - - - | 2 | 2 | 3 | 2 | | | 9 |
| Paralysis - - - - - - - - - - | 1 | 1 | 1 | 1 | 1 | 1 | 6 |
| Hydrocephalus - - - - (acute 7, chronic 13) | 2 | 1 | 3 | 2 | | 12 | 20 |
| Carried over | 92 | 114 | 107 | 102 | 94 | 92 | 601 |

*List of Cases received under the Care of the Physician at the Universal Dispensary for Children, St. Andrew's Hill, Doctors' Commons, London; from January* 30 *to July* 31, 1817.

| DISEASES. | Number of Cases received in each Month | | | | | | Total Number |
|---|---|---|---|---|---|---|---|
| | Feb. | Mar. | April. | May | June | July | |
| Brought over - - - | 92 | 114 | 107 | 102 | 94 | 92 | 601 |
| Icterus - - - - - - - - - - | 4 | 5 | 7 | | 4 | 1 | 21 |
| Leucorrhœa - - - - - - - - | | 1 | 1 | | | | 2 |
| Tabes mesenterica - - - - | 12 | 6 | 4 | 3 | | | 25 |
| Rachitis - - - - - - - - - - | | 1 | 3 | | 2 | 12 | 18 |
| Vermes - - - - - - - - (lumbrici 12, ascarides 11, tænia 4) - - - - | 9 | | 5 | 8 | 5 | | 27 |
| Hydrops - - - - - - - - - - | 1 | 2 | 1 | 1 | 1 | | 6 |
| Eruptiones variæ sine febre | 13 | 5 | 6 | 6 | 16 | 1 | 47 |
| Eruptiones cum febre - - | | 2 | 10 | | 6 | | 18 |
| Erysipelas - - - - - - - - - | 2 | 3 | 1 | 3 | 1 | | 10 |
| Lepra - - - - - - - - - - - - | | | | | | 2 | 2 |
| Bullæ - - - - - - - - - - - - | 1 | | | | | | 1 |
| Elephantiasis - - - - - - | 1 | | | | | | 1 |
| | 135 | 139 | 145 | 123 | 129 | 108 | 779 |
| Surgical cases - - - - - - | 38 | 23 | 42 | 51 | 31 | 19 | 204 |
| Totals. | 173 | 162 | 187 | 174 | 160 | 127 | 983 |

| | |
|---|---|
| Whereof have been cured - - - - - - - - - - | 739 |
| Have died - - - - - - - - - - - - - - - - | 25 |
| Are upon the books, and under cure - - - - - | 219 |
| | 983 |

Of the twenty-five children as above, four died of measles, and were under five years of age; two of pneumonia, and were under four; six of colliquative diarrhœa, and were under two; three of hooping-cough, of whom one was nine months old, and the other two under three years of age; two of convulsions, and were under two; one of phrenitis, who was seven years of age; four of hydrocephalus, and were under three; and three of tabes mesenterica, under two.

The list of cases shews that the prevalent diseases, at times, for five months, were measles, bilious remitting fever, mumps, inflammatory sore throat, inflammation of the brain and lungs, scarlet fever, rheumatism, diarrhœa, inflammation of the bowels, cholera morbus, hooping-cough, hydrocephalus, catarrhal fever, jaundice, nettle rash, and St. Anthony's fire.

*Total number of Admissions from the Opening of the Dispensary, June 24, 1816, to July, 1817.*

| | |
|---|---|
| Total cured and relieved - - - - - - - - - - - - | 1490 |
| Total died - - - - - - - - - - - - - - - - - - - | 39 |
| Total inoculated for cow-pox - - - - - - - - - - | 70 |
| At this time upon the books, as irregulars and under cure | 397 |
| | 1996 |

| | | |
|---|---|---|
| Total number admitted under the Physician - | 1674 | |
| Total number admitted under the Surgeons - | 322 | |
| | | 1996 |

From the date of the above Report to the end of February, 1817, influenza prevailed with the same degree of severity as in the months of November and December. Many of the worst cases occurred in the middle of February. At the attack, high pyrexia came on, with a quick respiration, severe cough, and a profuse discharge of acrid mucus from the pituitary membrane and the bronchiæ. In children of four and five months of age, the secretion was so excessive as to interpose a considerable obstacle to respiration, and to excite a perpetual desire to cough. This produced a spasmodic affection of the diaphragm and larynx, and a general irritation and perturbation of the whole system. In the state described, the interruption to respiration was frequent; a convulsive catch was perceptible in the muscles of the neck, and in the respiratory muscles: and in the worst instances of the disease, convulsive motions of the whole body ensued, with occasional suspensions of the breath, of some moments duration; from which state, however, the individual has been found to revive, and, oftener than once, to recover.

Now and then, inflammation of the lungs seemed to be the primary, and not the secondary disease. A cough has, in the first instance, attacked children, with difficult and apparently painful respiration, and severe pyrexia; whilst the countenance has been flushed, the eyes red, the tongue white, and the urine high-coloured. These symptoms frequently occurred about the middle of February, without any previous affection of the mucous membrane; but, when they did happen to be preceded by a catarrhal discharge, sneezing, and an irritable state of the lining of the trachea, then the inflammation of the lungs appeared to be a consequence, or termination of influenza: in other words, pneumonia succeeded, and became a part of the primary disease. When influenza passed into pneumonia, the child was incapable of sleeping, or making an inspiration, without being suddenly disturbed every few minutes by a fit of coughing, which was preceded by a sense of suffocation, and a stridulous sound in the voice, similar to the croup. The struggles occasioned by these symptoms produced a paroxysm of fever, of some hours' duration: the restlessness and agitation were extremely aggravated; the thirst excessive; the eyes were closed, and the pulse was rapid. In all these variations of this disease, the cough was a severe symptom, and the secretion of fluid into the trachea so copious as to compel the individual to make an effort, once in three or four minutes, to reject it. In general, a disease of this severity had been of many days' duration; and dissection proved, that the structure of the lining of the trachea, and even of the pituitary membrane, had undergone a change from inflammation, and that both were firmer and thicker than in their natural state. It also proved, that where these changes existed in the lining of the trachea, the lungs were also, in many instances, highly inflamed, and heavier than usual; and that they presented a structure in several spots, so dense and firm as to oppose considerable difficulty to that free circulation of the blood which is necessary for its purification, and for the purposes of health and life.

Until the middle of March, the above described disease continued to prevail with great severity; after which, the cases were comparatively few and mild. About this period the weather sat in very cold: no rain had then fallen during three or four weeks, and an easterly wind predominated. On the 15th, 16th, 17th, 18th, 19th, 20th, and on all the remaining days of the month, a number of children were brought into the Dispensary with severe pyrexia, a flushed face, rapid pulse, quick respiration, and a hard dry cough; the latter coming on, and having paroxysms of five or six minutes' duration, every half hour or hour. Here was a disease which denoted the existence of pulmonary inflammation in the first instance: it had no character whatever of influenza, though the symptoms were precisely similar to the secondary symptoms of that disorder. It came on with the well-marked diagnostics of idiopathic pneumonia, and without the precursory attendants of catarrh. Neither was the pneumonia of this season confined to infants at the breast; it indiscriminately attacked children from the age of three months to five and six years: in general, the danger was great. Unless the disease abated, on the third or fourth day the symptoms rapidly increased; the respiration became laborious and difficult: pyrexia was constant and severe; a comatose insensibility alternated with restlessness and great agitation; the eye was fixed, and had acquired a glassy appearance; the face became livid and swollen; the cough, though less violent, was frequent; and the child refused, indiscriminately, whatever was put to its mouth.

Of these cases, numbers yielded to copious bleedings by leeches, to brisk purgatives, to blisters applied to the sternum, to antimonials, and diluents: but it was necessary to resort to the most active means in the beginning of the attack; and, unless the bleedings were pushed to such an extent as to produce syncope, the inflammation still went on, and the child died about the tenth or twelfth day. Two small leeches applied to the sternum of a child, of a year old, have been frequently followed by such a discharge of blood as to produce paleness of the countenance, and cold perspirations about the face and neck, which have been succeeded by a diminution of pyrexia, an abatement of cough, and an evident relief in the breathing. Where the loss of blood has been considerable, children have recovered slowly. Still, no remedy was so effectual—no means afforded such prompt and permanent benefit.

Another description of disease, very common about the end of March, was a species of synocha, accompanied with gastric irritation, and pains indicative of congestion in the brain. Whole families of children, seven and eight in number, were brought into the Dispensary. Some complained of pain in the head and thirst; others complained of pain and tenderness in the præcordial region, and of sickness: all had high pyrexia. Some of the children were comatose; and upon being roused, to give an answer to their parent's questions, said, they had pain in the head. To these, the light seemed to give additional sufferings; the conjunctiva was slightly red, and the pupil dilated, but irritable upon being exposed to the light.

This affection of the head yielded to brisk purgatives, repeatedly administered; to local bleedings in the temples, and to blistering. It appeared to be of little service to give antimonials; but small doses of the submuriate of mercury were given with evident advantage, after the treatment above mentioned had been first carried into effect.

In those cases in which gastric disease predominated, the tongue was always furred; nausea and vomiting urgent, and the child could not suffer pressure on the præcordial region. The pyrexia was equally high as in those children who suffered from congestion in the brain. In either disease, the Reporter believes the pyrexia, or species of synocha, which prevailed, was merely symptomatic of organic disorder in the stomach, or in the brain. It did not abate till the functions of these organs were restored; and seemed to keep pace with, and to depend upon, the degree of derangement in them. Brisk purgatives, absorbents, saline diaphoretics, and fomentations, proved most efficacious. It was always necessary to excite a slight intestinal secretion for some days after the first stages of the disease had passed by. As the whole of these affections, variable as they were in their character and appearance, evidently originated in an inflammatory diathesis—in an increased vascular action—evacuants were liberally employed, and generally found to be efficacious.

In the course of February and March, many cases of jaundice occurred in children of three, four and five years of age, who had been very healthy till within a few days before they were brought to the Dispensary, to be treated for this complaint: slight pyrexia accompanied the disease. The skin was dry, of a reddish yellow hue, and the pulse quick. The children had no appetite for their food; the alvine discharge was trifling and difficult, the head painful, and the eye heavy.

The Reporter has frequently observed considerable hepatic derangement in children, from very slight causes. Even when this has not been evident, in the yellow tint of the skin, and the conjunctiva, it has been confirmed by inspecting the fæces, which have generally denoted a want of bile. At this time, the children were commonly peevish and irritable, had a morbid colour on the face, a darkness under the eyes, and a frequent inclination to pass their urine. By a long continuance of easterly winds with dry weather, the functions of the liver, in children, were sensibly influenced. Irritation from teething, or from worms, or from acrid substances in the primæ viæ, was always followed by hepatic derangement. The bile was separated from the blood, but it did not pass into the biliary ducts, or, at least, only in a certain portion, another portion having been absorbed and carried into the circulation. In the instances, however, of jaundice, which occurred at this season, it must be admitted, that *one* common cause was to be sought for in explanation of the production of the disease.

During the prevalence of the cold dry atmosphere of the same season, children were promiscuously attacked with inflammation of the lungs, the bowels, and the brain, accompanied with fever of the nature of synocha; others had jaundice with hepatic inflammation; and a great many more were attacked with cynanche, parotidæa, and with inflammatory enlargements of the submaxillary glands. Cynanche parotidæa was equally prevalent in the months of winter as in March. During all the cold months, it neither abated of, nor increased in, its severity: the pyrexia was always considerable, and the inflammatory action often continued till the third week.

The cases of cholera morbus were numerous and severe. Dyspeptic symptoms remained for weeks after: and diarrhœa, without pain, frequently succeeded an attack of cholera morbus; to remove which, astringents, tonics, and a change of air, were requisite. Absorbents availed but little. Hæmatoxylum and catechu were the most serviceable. Enteritis seldom followed cholera morbus; but the childrens' recovery was greatly retarded by diarrhœa.

One of the most formidable diseases which appeared, was an affection of the brain, of an inflammatory nature, attended with coma, redness of the conjunctiva, synocha, and an oppressed pulse. It was similar to the disease which was noticed, as having been of occasional occurrence, in the winter. The child could scarcely raise its head, was always restless, and started in its sleep; the bowels were torpid, and the urine was high coloured and small in quantity. This disease was idiopathic, and originated, apparently, in direct congestion of the vessels of the brain. It was a modified determination of the inflammatory diathesis, which so evidently characterised every complaint, of an acute description, which was brought, at this season, into the Dispensary. The nearer the child was the first year of age, the more severe was the attack; and if dentition happened to be in progress when the infant was assailed with this disease, the symptoms were of the most alarming kind. The Reporter could not discover any difference between this disease and the apoplexia hydrocephalica of Cullen, or acute hydrocephalus: the same kind of pulse existed, similar torpor of the bowels, coma, and dilatation of the pupils. No treatment was so efficacious, in averting the fatal tendency of this complaint, as copious bleeding by leeches, as alvine evacuations by brisk purgatives, and as a free discharge of serum by the application of blisters to the top of the head. Evaporating lotions to the neck and head were also serviceable, but the application of cold to the head had no beneficial effect.

The Reporter has always been liberal in prescribing topical bleedings in all acute diseases of the brain, trachea, and lungs, from a conviction that children sustain much better all evacuations of this kind, than those occasioned by the repetition of active purgatives. Neither did children experience such a permanent exhaustion from the loss of blood, as from copious watery secretions from the bowels. In the next place, it is an important practical fact, that diarrhœa was never so frequent among those children who had been previously bled by leeches, for the removal of any of these inflammatory diseases of the season, as those who had undergone another mode of treatment on the same account.

At the latter end of March, and in the beginning of April, several cases of acute rheumatism were admitted under the Reporter, in children of three, four, five, six, and seven years of age, and upwards. In many of these patients, so strongly marked was the inflammatory diathesis, that not only were the tension and swelling of the joints, with a slight efflorescence in particular spots around, distinctly observed, but even a redness of the conjunctiva; and in some of these children, the tonsils were at the same time inflamed, and the submaxillary glands enlarged and painful. Severe, however, as this disease was, it soon became mild and tractable by topical bleedings, purgatives, and saline diaphoretics.

In other patients, the muscles of the head and neck were principally the seat of rheumatism: the pyrexia in these cases was slight. This modification of rheumatism was confined to children of eight, ten, and twelve years of age. Blisters and diaphoretics usually carried it off. It was a slight disease; and although the head and temples partook of pain, there was no coma, nor any other symptom which could lead the practitioner to confound it with that organic affection of the encephalon, which has been noticed before

The cases of angina in this month were few, and such as did appear were mild, and without scarlatina. The reigning glandular disease was cynanche parotidæa. In many instances it was idiopathic, and totally independent of any enlargement of the neighbouring glands, as the salivary gland alone partook of inflammation: but, in other cases, the salivary gland, and the submaxillary gland, of the same side, both inflamed; and not unfrequently a chain of diseased glands extended from the cheek to the sternal extremity of the collar-bone.

In April, no cases of simple continued fever, or synocha, occurred, without the presence of local pain in the head, chest, or abdomen; neither did the constitution tend, in the instances of fever with local pain, to intermittent fever. A mild remitting bilious fever, of irregular paroxysms, was seen a few times; but it was preceded by bilious vomitings and diarrhœa. Hepatic derangement seemed to be the immediate cause of this fever, which abated as soon as the functions of the liver were restored. But not exactly in this fever, as in cholera morbus, did the symptoms subside, did the paroxysms cease, in proportion as the tone of the stomach and bowels was restored, and the irritability removed. When the first impressions upon these organs was overcome, still a fever of irregular type continued; and in the interval of the paroxysms, the head was painful, the skin dry and of a yellowish tint, the tongue white, the urine high coloured and small in quantity. During five or six days, these symptoms continued. A slight abatement of them took place in the morning; and a slight exacerbation at the approach of evening. Purgatives, consisting of the submuriate of mercury and rhubarb, of sulphate of magnesia, and the carbonate of magnesia, with saline diaphoretics, were very serviceable. Blisters to the right hypochondrium contributed to restore the functions of the liver. The subcarbonate of soda, dissolved in aniseed or mint-water, with a few drops of the tinct. lav. co. was a very useful medicine, given in the interval of the purgatives.

Some of the children, who had been attacked with this fever, had anasarca afterwards. Two died under it.

The first part of this Report shews, that the measles prevailed with great severity in the metropolis, in the middle of last winter. But this disease was also very formidable in April and May. A very large proportion of bad cases, in reference to the number of patients admitted with the measles, were brought into the Dispensary the latter end of March, and in April, and in May. When the weather continued dry and fine, the measles were milder; but when it became warm and moist, there was an evident aggravation in the character of the disorder. Independently of the measles having attacked, in this last state of the weather, with the usual diagnostic symptoms of pyrexia, hard cough, a red watery eye with swollen lids, vomiting, coma, &c. which, in the ordinary course of this complaint, abate on the third or fourth day, a secondary affection succeeded the primary attack, with symptoms of the most urgent nature. These were a severe cough with difficult and laborious breathing, intense pyrexia, shooting pains in the track of the small intestines, delirium, frequent inclination to vomit, a swollen and purple appearance of the face, excessive restlessness, and in some instances diarrhœa. Hoarseness usually prevailed throughout the stages of this secondary disease. The expectoration was trifling, but now and then intermixed with blood. The irritation along the trachea was extremely distressing; thirst incessant, and the tongue covered with sordes. The constitution of the child now sustained more extensive ravages than it had done from the immediate effects of the specific contagion which it had previously imbibed. It was not now a disease that exhausted all its action upon the pulmonary organ and the membrane of the trachea, but a secondary affection, that excited inflammatory pains in the bowels, irritation in the stomach, congestion in the lungs and brain; a disease that diffused a high inflammatory action over the whole system, more violent in its character than the primary disease, and more fatal in its effects. If the child recovered from an attack of this kind, extreme debility succeeded; and it was extenuated and hectic for many weeks. If the child died, it was comatose for three or four days before its death. Nothing roused it but the frequency and violence of the cough; the breathing was extremely difficult; diarrhœa and convulsions concluded the scene.

The Reporter is of opinion, that this secondary affection has frequently had its source in an inefficient treatment of the primary disease, in premature exposure to the weather, and in the too early administration of nutritious food. The extent of the eruption on the surface of the body was no criterion of the severity of the primary disease; neither was its duration there critical with respect to its solution. A slight eruption has been often preceded by severe symptoms, and succeeded by an intractable secondary affection; whilst a copious eruption has often appeared with very mild precursory symptoms, and yet been followed by a secondary attack of great violence. Nay, although the eruption has been very profuse, the child has frequently recovered without difficulty or relapse. In all the cases which came under the Reporter's notice, he does not remember to have seen one with the putrid diathesis described by authors. Petechiæ, it is true, have appeared; but they were neither of a livid or black colour, but literally red, and indicative of the highly inflammatory; and not of the putrid diathesis. In the first stages of a very severe kind of measles, little red spots, which have radiated as a star from a minute speck, the result of extravasation, have been observed. In the progress also of the disease, in the secondary stage, whilst the patient has been distressed with inflammatory pains in the intestines, with acute pains in the head from congestion, petechiæ of a similar kind have broken out upon the breast, arms, and legs. They never occurred but in cases of extreme severity, and did not indicate the existence of a putrid diathesis. After having had recourse to evacuations, particularly by bleeding and blistering, they disappeared under the use of diaphoretics; and where such evacuations had been adopted in the beginning of the disease, to a sufficient extent, the Reporter does not recollect ever to have seen any petechiæ; hence it may be concluded, that the appearance of these spots was an infallible indication, among some others, of the existence of a very strong inflammatory diathesis.

Erysipelas and urticaria were very frequent towards the end of March. The former was a severe disease, and attended with synocha. Two or three days before the eruption came out, the children had a fever of the description of synocha, with pain in the head and vomiting. The eruption spread itself principally on the neck, body, and extremities. The vesicles were large, with extensive efflorescence, and a thickening of the cellular membrane around. Small doses of sulphate of magnesia with antimonial wine, and saline diaphoretics, usually carried off the disease, after the previous exhibition of one or two active purgatives with submuriate of mercury and scammony. A slight opiate at night was also serviceable.

Some cases of a peculiar eruption, consisting of small pustules in clusters, with considerable efflorescence around each group, were brought into the Dispensary the beginning of April. The group was of varied form, sometimes oval, then round, and in other instances resembled in shape a truncated square. The pustules were distinct in several cases, and confluent in others.

This eruption attacked some children, whilst urticaria and erysipelas made their appearance in other children. The back, the neck, the arms, the legs, and body, were promiscuously the seat of the first kind of eruptive disorder. One child had two large groups upon the neck, which appeared soon after an enlargement of the glands of the same side, from cold. The pyrexia which accompanied the eruption was slight; the functions were but little disturbed, and the stomach was free from irritation.

During the prevalence of the cold dry weather, in the beginning of April, many patients were admitted with pneumonia, and many also with a harsh sonorous cough, and stridulous respiration with pyrexia, resembling a slighter kind of croup. The cases of pneumonia were all strikingly marked. They were not preceded by catarrhal symptoms, nor by the croupy cough with which many children were attacked. A short laborious breathing, a hard violent cough, high pyrexia, flushed countenance, a rapid pulse, came on at the beginning, and continued eight or ten days, about which time the disease began to subside, or symptoms threatening suffocation supervened. The treatment here, as in other acute disorders which attacked the chest, consisted in the early adoption of bleeding by leeches, of blistering, and in the use of purgatives and diaphoretics.

Three cases of genuine croup occurred in children at the breast. The symptoms were all severe, but yielded in a few hours to bleeding by leeches, ad deliquium, to blisters, evaporating lotions applied to the neck and throat, nauseating doses of a solution of tartarized antimony, and to a brisk dose of the submuriate of mercury and jalap. The Reporter found it useful to excite occasional vomiting in croup, by tickling the fauces with a feather, or the end of the finger. Unless the child was frequently urged by these means to make an effort to throw up the mucus which forms, in such large quantities, in cases of this kind, in the trachea and the bronchial tubes, a suffocative irritation and cough increased rapidly, and the danger of effusion was every hour to be apprehended.

Hepatic diseases were among the list of casualties of the season. Jaundice, with slight pyrexia, head-ache and diarrhœa, appeared in children, who had been admitted into the Dispensary with other complaints; and many fresh patients were also brought in, suffering with a similar disease. But jaundice was not always the first symptom of hepatic derangement. Even whilst under the influence of diarrhœa, some children were still evidently suffering from the disturbed state of the functions of the liver, though the skin was free from yellowness. Jaundice supervened in such, as a secondary symptom. Congestion in the liver is, probably, a frequent cause of illness in children. To this, during the infant state, this organ is naturally disposed only from its bulk, compared with the bulk of other organs. One reason, very likely, of the relief which children experience from copious alvine discharges, in many states of disease, is the sympathetic action which is communicated in this effort, from the intestines to the hepatic system, during the existence of such irritation, and a corresponding effect in an organ surcharged with blood, and in which the circulation is languid and incomplete!

The Reporter noticed in the months of April and May, some affections of the head in children of ten and twelve years of age, which resembled phrenitis. The symptoms were violent pyrexia, pain in the head with delirium, a rapid pulse, great agitation, inability to bear the light, redness of the conjunctiva, excessive thirst, suppression of the urine, spasmodic affections of the arms and legs, vomiting, and in general, constipation of the bowels. The disease was idiopathic, and denoted the presence of inflammation in the coverings of the brain. General bleedings, blisters to the head, and brisk purgatives succeeded in three instances of the disease. One case ended fatally, preceded by symptoms of effusion on the brain.

So late even as the end of May, the measles and the hooping-cough still formed a prominent feature in the diseases of this season. The hooping-cough promiscuously appeared as a primary disease; and if not as the sequel, at least, as an early successor of the measles. The most effectual treatment at the beginning of the hooping-cough, consisted in a lowering regimen, in preserving the bowels open with the submuriate of mercury and rhubarb, in administering slight diaphoretics combined with conium and ipecacuanha, and in employing leeches and blisters to the sternum. During the advanced stages of it, expectorants and opiates proved highly serviceable.

The throat was a frequent seat of disease in May and June. Cynanche tonsillaris was of general, cynanche maligna, of rare occurrence. Slight pyrexia accompanied an apthous appearance of the fauces, and constituted a specific disorder. The Reporter remembers to have seen the inside of the cheeks, and the whole of the fauces covered with apthous ulcerations; but, in these cases, the tonsils were neither tender nor enlarged, and deglutition was performed without the least difficulty. In many children, of eight and ten years of age, an eruption, resembling the red gum of infants, broke out upon the hands, breasts, and wrists, and was generally attended with an elargement of the glands of the neck. There was always pyrexia, which continued for four or five days, when the eruption became pale, and then dried up, leaving the child under a chronic inflammation of one or two glands, which not unfrequently ended in suppuration.

Frequent as cynanche parotidea has been through the whole of the year, no instance of metastasis, came under the Reporter's notice, among children. Two cases of acute inflammation of the testis, occurred in two plethoric healthy young men, in the Reporter's practice, and what is rather remarkable, the patients were brothers, one of whom was first attacked with cynanche parotidea, and then subsequent inflammation of the testis, a fortnight from the commencement of cynanche parotidea, of which the patient recovered after three weeks confinement. The brothers lived and slept together. Three weeks after the recovery of the first, the second brother was attacked with cynanche parotidea, in the same manner as the first. In a few days, the glandular inflammation began to subside, but before it had disappeared from the cheek, inflammation attacked the testis, and confined him also three weeks, to the house, as it had done his brother. Each had severe pyrexia, and required topical and general bleedings, and brisk purgatives. Neither recovered of this secondary affection for many weeks.

In May and June, numbers of children, from five to ten years of age, were brought into the Dispensary, labouring under high pyrexia and pungent pain in the side, with cough, and shortness of breathing. Some were admitted, with head-ache, pyrexia, vomiting, tension and uneasiness of the abdomen. Others had cholera morbus, or enteritis. Others again, came with a slight remitting bilious fever, which was attended with a copious vomiting of bile, nausea, a furred tongue, streaked with yellow sordes; considerable irritation, and drowsiness. The face was generally flushed, the skin of a yellow tint, and very hot. Towards evening, the exacerbation of the disease was remarkable; and the bowels throughout every stage of the fever, were torpid. In the few instances of this disorder, in which diarrhœa took place, the symptoms were mild. If it occurred in the progress of the fever, or towards its termination, it was critical. In the beginning of the fever, diarrhœa seemed to moderate the symptoms, and to shorten their duration. The primæ viæ undoubtedly sustained considerable irritation, even from the commencement of this fever, but the liver was the organ from whence the disease might be said to proceed. The secretions of this organ were totally changed; the bile was thin and acrid, and in excess. The tension of the right hypochondrium indicated congestion; and the yellowness of the skin was an evidence, that the bile did not all pass through its natural ducts, and that obstruction existed in the extreme vessels. The most successful treatment was that which excited the liver to new actions; to the discharge of all viscid and unhealthy bile; to a free circulation; and to the secretion of a natural fluid. Saline and mercurial purgatives, and blisters to the right hypochondrium accomplished these objects best. Absorbents fulfilled the second intention of correcting the acrimony of the fluids in the primæ viæ, and of allaying irritation in them. Diluents without any admixture of stimuli, were very useful.

In a variety of cases, the functions of the liver became suspended from other causes; and from none, oftener, than from a dyspeptic, languid state of the stomach, which was generally brought on by the administration of improper food, and food in excess. Before, however, the liver evidently ceased to secrete bile of a proper quality, and in sufficient quantity, there were many slight symptoms of indisposition to be detected in the children, such as paroxysms of fever, which came on and went off, at irregular periods, without any sensible crisis; heat and dryness in the palms of the hand and soles of the feet; a fulness in the epigastric region; a peevishness of temper; lassitude; softness of the skin; and an inclination to eat at all hours. After a duration of these symptoms for several days, (symptoms scarcely paid any attention to even by parents) the fæces changed their colour, and became very offensive; and unless the state of the digestive organs was then improved, a diarrhœa succeeded, in which the stools were voided with force, suddenly, and with an admixture only of thin yellowish bile. If, in the first instance, this diarrhœa was observed to relieve some of the symptoms just described, it brought on others which were more urgent and distressing than those it had abated. Except during the period of dentition, this discharge was rarely critical. High pyrexia and irritation never failed to accompany diarrhœa after it had continued three or four days. By the early administration of aperient medicines, diarrhœa was frequently averted; and its removal, after it had once been established, promoted by the occasional interposition of doses of rhubarb and submuriate of mercury; absorbents alone seldom sufficed to stop diarrhœa.

It required catechu and hæmatoxylum, the latter of which was by far the most efficacious medicine. A very material object to keep in view, both for the preservation of childrens' health, as well as for its restoration from disorders of the digestive organs, was the correction of the secretions of the liver, which were invariably vitiated, defective, or in excess, during the months of summer. One of the most frequent diseases, from this cause, was diarrhœa, which, however, rarely occurred after the epoch of dentition, in such children as had been purged from time to time, and to whom slight tonics, combined with aperients, had been exhibited. But it was still evident that the liver was, in a variety of cases, the seat of secondary disease. The digestive organs, in a disordered state, communicate a morbid irritation to the liver, an action which was productive of the same results as if this organ had been primarily affected. The Reporter invariably noticed this, in those children who had large bellies, whose skins were soft and pale, and whose joints had a ricketty appearance. Whenever the functions of the liver were so materially disturbed, as to be evident upon a slight inspection of the child's health, then irregular paroxysms of fever attended, which had the effect, at each return, of heightening the tendency to hepatic obstruction. The first step to a cure in all these cases, was to restrict the child in its food ; to suffer only such a quantity to be given, as could be easily digested ; and food of such a quality, as neither excited irritation, nor afforded too great a quantity of nutritive substance. A second means consisted in the employment of gentle aperients and tonics ; and when the habit had been thus altered, in the removal to a good salubrious air. By this plan, the digestive organs acquired energy, the functions of the liver were restored, and the child became healthy. Under other circumstances of irritation in the primæ viæ, eruptions of a mixed and varied nature broke out upon the legs, arms, body, and face, and not unfrequently upon the scalp. Slight pyrexia occasionally accompanied these eruptions, but more commonly the health was good and free from general disturbance. The tongue was rather white and furred ; but the fæces were tinctured with bile of a healthy colour.

Such was the state of the children, who were brought to the Dispensary in the early stages of irritation of the digestive organs, in which the surface had participated. If no assistance was given at this period, the children afterwards became dyspeptic, and had diarrhrœa; and from having been plump, robust subjects, became weak, feeble, and emaciated. An irregular excitability existed in the bowels of such patients; a portion of the intestines acted freely in them, and discharged in copious quantities, a watery fluid: whilst, another portion remained indolent, and inclosed in the plicæ fæculent substances, which were productive of perpetual irritation, and proved a source of successive paroxysms of fever, and successive attacks of diarrhœa. In all these instances, drastic purges did harm. They exhausted the patient, without removing the disease. By promoting a slight action, on the contrary, in the bowels, for a fortnight or longer, the removal of these substances was accomplished, and the bowels restored in all their parts to their proper functions. Slight tonics, and preparations of sulphur with soda, were also particularly useful.

Another consequence of neglected disease of the digestive organs, was mesenteric obstructions, which progressively came on after a long continuance of the before mentioned symptoms. Thus was an incurable atrophy finally established.

During the months of June and July, a great many cases of cholera morbus, enteritis, and fever of a remitting nature, with yellowness of the skin and conjunctiva, were brought into the Dispensary. Diarrhœa, scarlatina, hooping-cough, and measles were also met with. Scarlatina was extremely mild. But the most severe disease of the summer months, was an inflammatory affection of the brain with synocha, which indiscriminately attacked children of all ages, to the tenth and twelfth year. A vast many cases of this kind were brought in during the continuance of the hot days in June. Chronic and acute hydrocephalus have been frequently seen. Highly ricketty children, and others, those whose digestive organs had been much impaired, and debilitated, were attacked with convulsions; which, at one time, appeared to originate in pressure on the brain from water, and at another time to be primary, and terminate by producing such a result. A number of these patients entirely recovered by the use of purgatives, tonics, and the volatile alkali. Towards the latter end of July, a few children at the breast were presented at the Dispensary with pneumonia, which had been preceded by catarrhal symptoms.

It is as interesting as it is important, to notice the material difference that exists in the character of diseases in children, at various seasons of the year, and even in the same constitutions, at the different epoch of childhood. The Reporter has observed some of the very same children, who have in the last few months been attacked with a kind of bilious remitting fever, in former seasons experience repeated attacks of synocha, to which they now seem no longer subject. Neither has it occurred to the Reporter to meet with those cases of cynanche maligna in this class of patients, which he remembers to have seen in former years. Those which have fallen under his observation have been few, and comparatively mild. The general character of fever, not only in children, but also in adults, has certainly experienced an essential alteration. Within a few years, diseases, on the contrary, of the intellectual functions, have become more severe, and more frequent.

